# The burden of obesity in women of reproductive age and in pregnancy in a middle-income setting: A population based study from Jamaica

**DOI:** 10.1371/journal.pone.0188677

**Published:** 2017-12-13

**Authors:** Lovney Kanguru, Affette McCaw-Binns, Jacqueline Bell, Novie Yonger-Coleman, Rainford Wilks, Julia Hussein

**Affiliations:** 1 USHER Institute of Population Health Sciences and Informatics, School of Medicine and Veterinary Medicine, University of Edinburgh, Scotland, United Kingdom; 2 Department of Community Health and Psychiatry, University of West Indies, Mona, Jamaica; 3 NHS Grampian, Scotland, United Kingdom; 4 Caribbean Institute for Health Research, University of West Indies, Mona, Jamaica; 5 Independent Maternal Health Consultant, Scotland, United Kingdom; Loyola University Chicago, UNITED STATES

## Abstract

**Introduction:**

Obesity is rising globally and is associated with increased risk of adverse pregnancy outcomes. This study aims to investigate overweight and obesity and its consequences among Jamaican women of reproductive age, particularly development of diabetes, hypertension and the risk of maternal death.

**Materials and methods:**

A national lifestyle survey (2007/8) of 1371 women of reproductive age provided data on the prevalence of high BMI, associated risk factors and co-morbidities. A national maternal mortality surveillance database (1998–2012) of 798 maternal deaths was used to investigate maternal deaths in obese women. Chi-squared and Fisher exact tests were used.

**Results:**

High BMI (> = 25kg/m^2^) occurred in 63% of women aged between 15 and 49 years. It was associated with increasing age, high gravidity and parity, and full time employment (p<0.001). Of those with high BMI, 5.5% were diabetic, 19.3% hypertensive and 2.8% were both diabetic and hypertensive. Obesity was recorded in 10.5% of maternal deaths, with higher proportions of deaths due to hypertension in pregnancy (27.5%), circulatory/ cardiovascular disorders (13.0%), and diabetes (4.3%) compared to 21.9%, 6.9% and 2.6% respectively in non-obese women.

**Conclusions:**

This is one of a few studies from a middle-income setting to explore maternal burden of obesity during pregnancy, which contributes to improving the knowledge base, identifying the gaps in information and increasing awareness of the growing problem of maternal overweight and obesity. While survey diagnostic conditions require cautious interpretation of findings, it is clear that obesity and related medical conditions present a substantial public health problem for emerging LMICs like Jamaica. There is an urgent need for global consensus on routine measures of the burden and risk factors associated with obesity and development of culturally appropriate interventions.

## Introduction

Obesity among women of reproductive age varies across low and middle-income countries (LMIC) from 3.4 to 73.7% [1,2]. These women are more susceptible to pregnancy complications such as pre-eclampsia, wound infections, anaesthetic complications, miscarriages and cardiovascular problems in pregnancy and consequent maternal death. This obstetric risk is potentially higher in low resource settings where specialized care for obstetric and medical problems associated with obesity may not be optimal.

LMICs are currently undergoing an epidemiological transition, with a shift in disease patterns from communicable to non-communicable conditions. Globalization and urbanization are recognized drivers of this transition, mediated by changes in lifestyles and habits [3]. Behavioral changes include consumption of unhealthy foods, alcohol misuse and physical inactivity, all primary risk factors for becoming overweight and obese [4]. People in middle-income countries are reported to have consistently increasing levels of fat intake since the 1980s [5].

With 99% of global maternal deaths occurring in LMICs, and with suboptimal rates of decline, maternal mortality remains a major public health concern[6].While most deaths (72%) are due to obstetric or direct causes such as postpartum bleeding, puerperal infections, eclampsia, abortion and obstructed labor, medical or indirect causes account for almost a third of all maternal deaths [7]. Such conditions may develop or are aggravated by the physiological effects of pregnancy [8], with some pre-existing conditions first getting diagnosed during pregnancy or the puerperium. These include non-communicable diseases (NCDs) such as cardiovascular conditions, diabetes and anemia, and communicable diseases such as HIV, malaria and tuberculosis. The contribution of some of these medical conditions is well known. For example, HIV is estimated to account for 2.6% of all maternal deaths globally [6], while anemia contributes to 12.8% of all maternal deaths in Asia and 3.7% in Africa [9].Less is known about the contribution of other medical conditions to pregnancy related morbidity and mortality in LMICs, such as obesity and associated diseases. Additionally, more than 50% of indirect causes of maternal deaths are not routinely classified [7] and tend to be reported together, without identifying the specific underlying conditions. While previously thought of as diseases associated with old age, many NCDs are increasingly being reported in younger populations including women of reproductive age. These proportions are expected to exponentially increase by 2030 for many LMIC [3,8].

Jamaica has had some success in reducing maternal deaths, however the decline has been slow, from 98 per 100,000 live births in 1990 to 80 per 100,000 in 2013 [6], with indirect deaths accounting for an increasing proportion of these deaths [10]. This is despite the high uptake of maternity care services. For example, 87% of women attend at least 4 antenatal visits [11], and 96% are attended by skilled health professionals in hospitals [12]. While public sector uptake of postnatal care has fluctuated between 67% and 74% during the last ten years [11], immunization coverage is high, suggesting that postnatal services are being accessed in the private sector.

Our objectives were to investigate the association of overweight and obesity with:

1. The presence of diabetes and hypertension among women of reproductive age
2. The causes of maternal deaths, especially those due to circulatory/ cardiovascular, endocrine and metabolic disorders including direct (obstetric) complications of pregnancy.
3. Perinatal outcome among women who died during pregnancy, childbirth or the puerperium.

## Methods

### Ethical approval

The University of West Indies-Mona [ECP192-12/13) and the Ministry of Health in Jamaica provided ethical approval [S&R/ERP/7).

### Data sources

Two data sources were used: the most recent Jamaica Health and Lifestyle Survey (JHLS, 2008) and the Jamaica Maternal Mortality Surveillance (JMMS) database (1998–2012). The JHLS is a nationally representative survey that monitor lifestyle, health and behavioral patterns of the Jamaican population across different socio-economic groups and demographic characteristics. Elements include reproductive health, health-seeking behavior, medical conditions (including diabetes) and socio-economic indicators [13]. Physical measurements (for example blood pressure and fasting blood glucose) and anthropometric measurements (including height and weight) are also collected. Data from 1,371 women of reproductive age (15 to 49 years) who participated in the 2007/8 survey were used in this study. About 4–5% of these women had given birth in the 6 months before the survey, thus were in the postpartum phase [13].All women participants in the survey needed to indicate that they were not pregnant in order to be eligible.

The JMMS is a national database of routinely identified and reviewed deaths in women of reproductive age in which a pregnancy or birth was reported within a year of the death. Surveillance officers monitor hospital records (health management information system, autopsy reports, death registers) while community teams report on maternal deaths occurring outside health facilities. Verbal autopsies with the next of kin are conducted where possible. Some events however may be missed [14]. All reported maternal deaths are reviewed by a multi-disciplinary team and classified as obstetric (direct causes), non-obstetric (indirect causes) and coincidental (incidental causes). Late maternal deaths and coincidental deaths, while reviewed, are excluded from national MMR estimates. The database captures demographic data as well as use of and access to health services. The maternal mortality surveillance system in Jamaica became fully operational in 1998 [15]. From1998 to 2012, 798pregnancy related deaths (617 maternal, 127 late maternal, 39 coincidental, 15 unclassified) were captured in the JMMS database and used in this study.

### Outcome and exposure variables

Women of reproductive age (WRA) who were obese were identified by using height and weight measurements from the JHLS to calculate body mass index (BMI). The BMI thresholds used for the three exposure categories were as follows: normal (18.5–24.99 kg/m^2^); overweight (25–29.99 kg/m^2^) and obese (more than or equal to 30 kg/m^2^)[16]. In this paper, we use the term ‘high BMI’ for data originating from JHLS to include both the overweight and obese categories. The diagnosis of diabetes and hypertension in JHLS relied on single clinical measurements done during the survey, so are unlikely to conform to international standards for diagnosis. Different forms of diabetes or hypertension could not be differentiated. The Jamaica guideline on diabetes stipulates screening pregnant women using 50g glucose challenge test with those positive undertaking 2h (100g) oral glucose tolerance test [17]. Positive diagnosis is made when on two or more occasions the fasting blood glucose ≥5.3mmol/L, 1h ≥10mmol/L, 2h ≥8.6mmol/L and 3h ≥7.8mmol/L. For hypertension the national guideline stipulates using the criteria of normal (<120/<80mmHg), pre-hypertension (120-139/80-89mmHg), stage 1 (140-159/90-99mmHg) and stage 2 (≥160/≥110mmHg) for all adults 18 years and above [18]. Diagnosis of pregnancy induced hypertension is confirmed with diastolic pressures of 90mmHg or more on two consecutive occasions 4 hours apart with no proteinuria. If proteinuria is present then pre-eclampsia is confirmed. JHLS socio-demographic covariates include age, education, employment, setting (urban/rural), gravidity and parity.

The JMMS database allows for the recording of up to three causes of death, the underlying cause of death that initiates the chain of events or complications leading to death, the intermediate condition arising from the underlying cause of death and the immediate cause of death, or the final assault before death. As the underlying and immediate causes of death were most consistently documented, these will be reviewed. Being overweight or obese was not listed as a cause of death, but could be recorded as a contributing condition on the medical certificate or as part of the clinical summaries compiled for the case review process. All conditions in the JMMS database are classified according to the condition as reported in the JMMS forms. These are completed by clinicians and pathologists, and reviewed for documentary evidence of overweight or obesity and combined into the variable ‘obesity’ in this study. Thus, BMI calculations during pregnancy or postpartum were based on clinical assessments/ judgments. The JMMS database classified the perinatal outcomes as: full-term live birth, preterm live birth, stillbirth, non-viable outcome or died undelivered. Social and demographic indicators in the database include age, setting (rural/urban), parity, gravidity and cause of death.

### Analysis

In the JHLS database preliminary analyses were conducted to identify any associations between potential risk factors and high BMI, diabetes and hypertension. Pearson chi-squared test, chi-squared test with continuity correction and Fisher’s exact test were used as appropriate. When statistical assumptions were not met, categories were combined. Co-morbidities between the three conditions of interest (high BMI, diabetes and hypertension) across the different age groups were investigated.

In the JMMS database the underlying and immediate causes of death for all maternal deaths in the period 1998 to 2012 were compared between the women classified as obese and those with no documented evidence of obesity. Conditions known to occur more frequently in the obese population (hypertension (including pre-eclampsia), circulatory disorders, diabetes mellitus, embolism and complications of anaesthesia) were reviewed separately and as a group. Cases were summarized according to the WHO ICD-MM guidelines [19]. Chi-square tests for associations between the causes of maternal death and obesity were conducted. Co-morbidities among the obese women were also investigated.

## Results

### Obesity and related conditions in women of reproductive age

Table 1 shows the socio-reproductive profile of Jamaican women of reproductive age captured by the 2007/8 JHLS. Over 95% of women have secondary or higher education, 52% are employed and 60% are urban residents. The majority (almost 80%) of the women had experience done or more live births.

**Table 1:** Percentage distribution of socio-demographic and reproductive variables in women of reproductive age and in those with (and without) high BMI, diabetes and hypertension, JHLS 2007/8.

Variable	All women (1371), N (%)	High BMI (>25kg/m^2^), N (row %)			Diabetes mellitus, N (row %)			Hypertension, N (row %)		
		With (N = 861)	Without	P value	With (N = 91)	Without	P value	With (N = 331)	Without	P value
Age				*<0*.*001*			*<0*.*001*			*<0*.*001*
15–19	154 (100%)	44 (28.6%)	104 (67.5%)		2 (1.3%)	152 (98.7%)		7 (4.6%)	146 (94.8%)	
20–24	184 (100%)	82 (44.57%)	97 (52.7%)		2 (1.1%)	182 (98.9%)		33 (17.9%)	151 (82.1%)	
25–29	198 (100%)	119 (60.1%)	76 (38.4%)		4 (2.0%)	194 (98.0%)		39 (19.7%)	159 (80.3%)	
30–34	224 (100%)	144 (64.3%)	69 (30.8%)		8 (3.6%)	216 (96.4%)		44 (19.6%)	180 (80.4%)	
35–39	210 (100%)	163 (77.6%)	43 (20.5%)		17 (8.1%)	192 (91.4%)		57 (27.1%)	152 (72.4%)	
40–44	202 (100%)	149 (73.8%)	50 (24.8%)		25 (12.4%)	176 (87.1%)		66 (32.7%)	136 (67.3%)	
45–49	199 (100%)	160 (80.4%)	34 (17.1%)		31 (15.6%)	168 (84.4%)		85 (42.7%)	114 (57.3%)	
Education				*0*.*688*			*0*.*087*			*0*.*151*
No schooling	3 (100%)	3 (100.0%)	0		0	3 (100.0%)		2 (66.7%)	1 (33.3%)	
Primary (includes basic school)	32 (100%)	22 (71.0%)	9 (29.0%)		6 (18.8%)	26 (81.3%)		5 (15.6%)	27 (84.4%)	
Secondary (includes junior high)	1031 (100%)	648 (64.5%)	356 (35.5%)		70 (6.8%)	960 (93.2%)		263 (25.5%)	768 (74.5%)	
Vocational/technical	118 (100%)	71 (61.7%)	44 (38.3%)		7 (5.9%)	111 (94.1%)		24 (20.7%)	92 (79.3%)	
Tertiary/college	141 (100%)	86 (63.2%)	50 (36.8%)		6 (4.3%)	135 (95.7%)		27 (19.1%)	114 (80.9%)	
Other	46 (100%)	31 (68.9%)	14 (31.1%)		2 (4.3%)	44 (95.7%)		10 (21.7%)	36 (78.3%)	
Employment				*<0*.*001*			*0*.*072*			*<0*.*001*
Unemployed	535 (100%)	324 (62.2%)	197 (37.8%)		38 (7.1%)	496 (92.7%)		141 (26.4%)	393 (73.6%)	
Full time	549 (100%)	391 (73.1%)	144 (26.9%)		44 (8.0%)	504 (91.8%)		143 (26.1%)	405 (73.9%)	
Part-time/seasonal/other	163 (100%)	112 (71.3%)	45 (28.7%)		6 (3.7%)	157 (96.3%)		41 (25.2%)	122 (74.8%)	
Student	122 (100%)	32 (26.9%)	87 (73.1%)		1 (0.8%)	121 (99.2%)		5 (4.1%)	117 (95.9%)	
Setting				*0*.*426*			*0*.*066*			*0*.*537*
Urban	818 (100%)	505 (63.7%)	288 (36.3%)		49 (6.0%)	768 (93.9%)		192 (23.5%)	624 (76.3%)	
Rural	553 (100%)	356 (65.8%)	185 (34.2%)		42 (7.6%)	511 (92.4%)		139 (25.1%)	414 (74.9%)	
Gravidity				*<0*.*001*			*<0*.*001*			*<0*.*001*
Gravida– 0	228 (100%)	82 (37.1%)	139 (62.9%)		5 (2.2%)	223 (97.8%)		9 (2.7%)		
Gravida– 1	262 (100%)	152 (59.6%)	103 (40.4%)		15 (5.7%)	247 (94.3%)		68 (20.5%)		
Gravida– 2	236 (100%)	151 (67.1%)	74 (32.9%)		11 (4.7%)	225 (95.3%)		65 (19.6%)		
Gravida– 3–5	501 (100%)	369 (75.3%)	121 (24.7%)		43 (8.6%)	458 (91.4%)		135 (40.8%)		
Gravida– ≥6	141 (100%)	107 (75.9%)	34 (24.1%)		15 (10.6%)	124 (87.9%)		41 (12.4%)		
Parity				*<0*.*001*			*0*.*004*			*<0*.*001*
0	282 (100%)	114 (42.1%)	157 (57.9%)		10 (3.5%)	272 (96.5%)		22 (7.8%)	260 (92.2%)	
1	284 (100%)	175 (63.2%)	102 (36.8%)		13 (4.6%)	271 (95.4%)		68 (24.0%)	215 (76.0%)	
2	253 (100%)	164 (67.2%)	80 (32.8%)		18 (7.1%)	235 (92.9%)		65 (25.8%)	187 (74.2%)	
3–5	444 (100%)	328 (75.4%)	107 (24.6%)		33 (7.4%)	410 (92.3%)		135 (30.4%)	309 (69.6%)	
≥6	107 (100%)	80 (74.8%)	27 (25.2%)		15 (14.0%)	91 (85.0%)		41 (38.3%)	66 (61.7%)	

The association between maternal characteristics and obesity is also shown in Table 1. Obesity is associated with increasing maternal age, gravidity and parity (p<0.001) and full time employment. There were no associations with education or urban-rural residence. The risk factor distributions were similar for diabetes and hypertension. However, caution should be taken in the interpretation of patterns for diabetes due to the smaller numbers involved.

**Fig 1:**
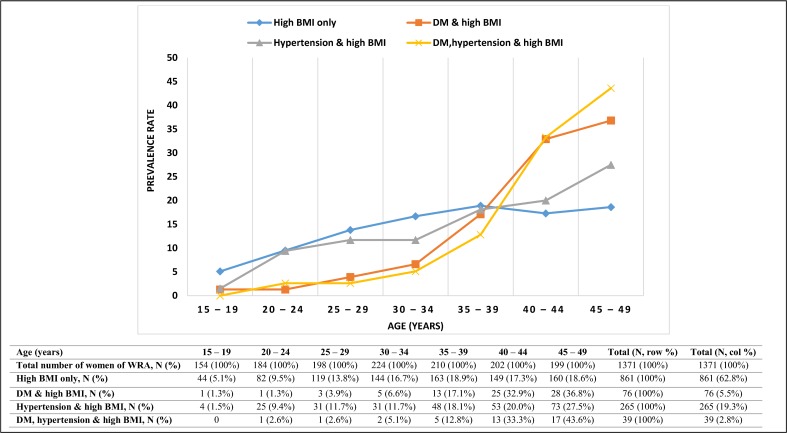
Age-specific prevalence of different combinations of high BMI, DM and hypertension among women of reproductive age (WRA) in the JHLS sample, 2007/8.

Fig 1 shows age specific prevalence of different combinations of high BMI, DM and hypertension among women of reproductive age. Overall, High BMI (> = 25kg/m^2^) occurred in 63% of women aged between 15 and 49 years. The prevalence of high BMI only, that is those with BMI exceeding 25kg/m^2^, increased with maternal age (up to 35–39 years) then plateaued afterwards. Of these women, the prevalence of diabetes was 5.5% (range 1.3–36.8%), of hypertension 19.3% (range 1.5–27.5%) and of both diabetes and hypertension 2.8% (range 2.6–43.6%). Most women with diabetes or hypertension were overweight or obese (84.4% of diabetics and 81.0% of those with hypertension; data not shown).

### Maternal deaths associated with overweight or obesity: Distribution and trends

**Fig 2:**
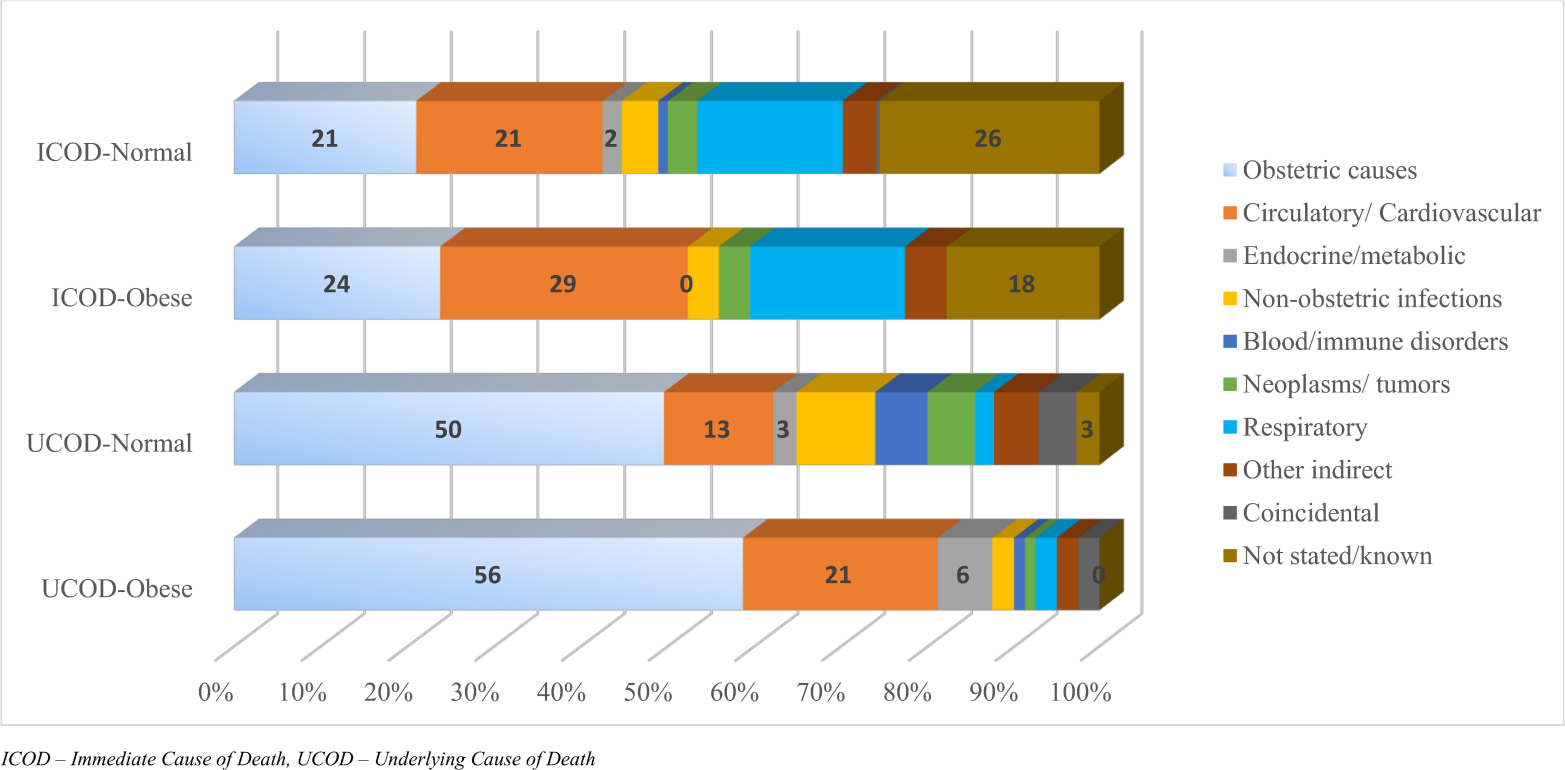
Immediate (ICOD) and underlying (UCOD) cause of maternal deaths related to high BMI (overweight or obesity).

A total of 798 pregnancy related deaths were recorded in the JMMS database between 1998 and 2012 (Table 2). Over this period, obesity was recorded among 84 maternal deaths (10.5%). Of these women, 83% were maternal deaths (direct/indirect causes that occurred during or within 42 days of the end of pregnancy) and 15% were late maternal deaths (that occurred within 43–364 days delivery). These proportions did not differ significantly from the 77% and 16% respectively observed among non-obese women. The three leading underlying causes of death (UCOD) among women with high BMIs included obstetric causes (56%), followed by circulatory/cardiovascular causes (21%), and endocrine/metabolic disorders (6.2%)(Fig 2). These three conditions were ranked 1^st^ (50%), 2^nd^ (13%), 8^th^ (3%) respectively among non-obese women.

**Table 2:** Maternal deaths among women classified as obese and otherwise, Jamaica maternal mortality surveillance database, 1998–2012.

Designation and study period		Direct/indirect < = 42 days	Late direct/indirect	Coincidental < = 42 days	Cause not known	Total
Designated as Obese	1998–2000	16 (80.0%)	4 (20.0%)	0	0	20
	2001–2003	31 (83.8%)	5 (13.5%)	1 (2.7%)	0	37
	2004–2006	10 (76.9%)	2 (15.4%)	1 (7.7%)	0	13
	2007–2009	9 (90.0%)	1 (10.0%)	0	0	10
	2010–2012	4 (100%)	0	0	0	4
**Sub-Total**		**70 (83.1%)**	**12 (14.5%)**	**2 (2.4%)**	**0**	**84**
**Not** designated as obese	1998–2000	114 (79.7%)	20 (14.0%)	3 (2.1%)	6 (4.2%)	143
	2001–2003	101 (73.7%)	29 (21.0%)	6 (4.4%)	1 (0.7%)	137
	2004–2006	117 (79.1%)	21 (14.2%)	6 (4.1%)	4 (2.7%)	148
	2007–2009	104 (73.8%)	26 (18.4%)	9 (6.4%)	2 (1.4%)	141
	2010–2012	111 (76.6%)	19 (13.1%)	13 (9.0%)	2 (1.4%)	145
**Sub-Total**		**547 (76.8%)**	**115 (16.1%)**	**37 (5.2%)**	**15 (2.1%)**	**714**
**Grand total**		**617 (77.3%)**	**127 (15.9%)**	**39 (4.9%)**	**15 (1.9%)**	**798**

We further examined in detail whether there were significant associations between women designated as obese or not with five conditions recognized as occurring more frequently among the obese population, (i.e. hypertension (ICD codes 011–016), embolism, complications of anaesthesia, circulatory/ cardiovascular disease and diabetes mellitus). Results of Table 3 shows that these five conditions were associated with 59.4% of maternal deaths among the obese population compared to 41.8% among the other women, especially hypertension (ICD codes 011–016) (27.5%), circulatory/ cardiovascular diseases (13.0%), embolism (10.1%) and diabetes (4.3%). The distribution of these conditions do not differ significantly when the non-obese group are compared with the obese group (p = 0.135). However, it differs significantly when compared with the direct causes, indirect causes and complications of care (p = 0.038), including coincidental causes (p = 0.035). No associations were found with late maternal deaths or coincidental deaths.

**Table 3:** Causes of death, comparing women designated in the JMMS database as obese or not obese^1^.

Cause of death	Designated as obese		Not designated as obese		P
**WHO maternal deaths^2^**	**N**	**%**	**N**	**%**	***0*.*038 (Χ***^***2***^_***3df***_ ***= 8*.*42)***
1. Complications associated with obesity	41	59.4	229	41.8	
- Hypertension (ICD codes 011–016)	19	27.5	120	21.9	
- embolism	7	10.1	48	8.8	
- complications of anaesthesia	3	4.3	9	1.6	
- circulatory/ cardiovascular	9	13.0	38	6.9	
- diabetes mellitus	3	4.3	14	2.6	
2. Other direct	17	24.6	176	32.1	
3. Other indirect	8	11.6	118	21.5	
4. Other complications of care	3	4.3	25	9.6	
Sub-total	**69**	**100**	**548**	**100**	
**Late maternal deaths^3^**	**Designated as obese**		**Not designated as obese**		***NS***^***4***^ ***(Fisher’s P = 0*.*19)***
1. Complications associated with obesity	4	33.3	35	31.0	
- Hypertension (ICD codes 011–016)	0	0	3	2.7	
- embolism	0	0	7	6.2	
- circulatory/ cardiovascular	3	25.0	18	15.9	
- diabetes mellitus	1	8.3	7	6.2	
2. Other direct	4	33.3	29	25.7	
3. Other indirect	3	25.0	48	42.5	
4. Other complications of care	1	8.3	1	0.9	
Sub-total	**12**	**100**	**113**	**100**	
**Coincidental^5^**	**Designated as obese**		**Not designated as obese**		***NS***^***4***^ ***(Fisher’s*** ***P = 1*.*00)***
1. Other indirect (neoplasia)	0	0	9	25.0	
2. External causes of death	2	100.0	27	75.0	
- unintentional injuries	1	50.0	10	27.8	
- intentional injuries	1	50.0	17	47.2	
- other	-	-	-	-	
Sub-total	**2**	**100**	**36**	**100**	
**All maternal deaths ^2^^,^^3^^,^^5^**	**Designated as obese**		**Not designated as obese**		***0*.*035 (Χ***^***2***^_***4df***_ ***= 10*.*46)***
1. Complications associated with obesity	45	54.2	264	37.8	
2. Other direct	21	25.3	205	29.4	
3. Other indirect	11	13.3	166	24.8	
4. Other complications of care	4	4.8	26	3.7	
5. Coincidental	2	2.4	36	4.3	
Grand total	**83**	**100**	**697**	**100**	

1 The term obesity includes overweight, obese and morbidly obese women

2 Direct and indirect deaths during pregnancy and up to 42 days post delivery

3 Direct and indirect deaths 43–364 days post delivery

4 NS–not significant

5 Accidents, violence, conditions not exacerbated by pregnancy (neoplasia, pregnancy incidental PM finding)

### Neonatal outcomes

**Fig 3:**
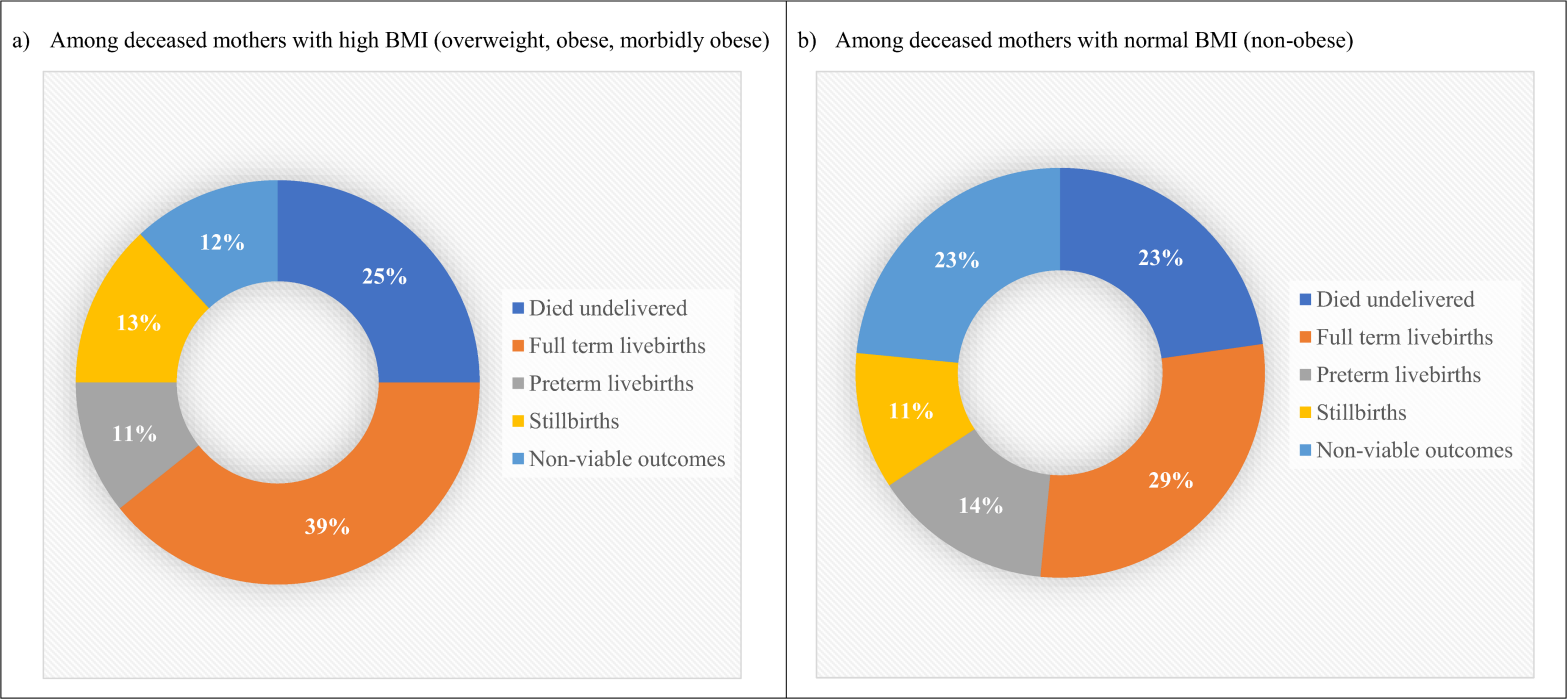
Percentage distribution of perinatal outcomes.

Fig 3 compares the perinatal outcomes among the two populations of maternal deaths in Jamaica. Among the maternal deaths associated with obesity (Fig 3A), approximately half of these women had a live birth (39% full-term, 11% preterm) which was not remarkably different from the non-obese maternal deaths (43%; 29% full-term, 14% preterm) (Fig 3B). Similarly, the proportion of mothers who died undelivered was not different at 25% and 23% respectively. There were however, slight differences found for non-viable outcomes (12% and 23%) but not stillbirths (13% and 11%) (p = 0.071).

## Discussion

Current estimates in LMICs of overweight and obesity in women of reproductive age range between 11 and 40% [20]and are rising [21, 22]. Indeed, the prevalence of overweight and obesity currently exceeds that of underweight in all regions of the world [23] although it is likely that subnational variations occur, for instance between rural and urban areas [21,24]. In sub-Saharan Africa, between a quarter and a third of urban women were overweight or obese and levels rose by nearly 35% between 1992 and 2005[21]. We found that 62% of Jamaican women of reproductive age had a high BMI, a prevalence similar to a study conducted in Chile [25] and consistent with global estimates from 2008 reporting levels of more than 70% in the Americas and Caribbean [22]. These surpass the levels generally seen in LMICs and even those in industrialized countries, which report prevalence of up to 59%[20,26]. Our study showed that obesity and overweight rose with increasing age and parity, a well-established association [27]. Improved education, socio-economic status (SES) and urban residence are known risks for obesity in LMICs [21,24,28].Despite lack of SES data in our dataset, full time employment (with implications of wealth and education) appeared to increase the risk of obesity. However, caution should be taken in interpreting this result.

Compared to the general population of women of reproductive age in Jamaica, those with high BMI had higher levels of diabetes and hypertension, while over 80% of women with these conditions were also overweight. Others have shown that overweight or obese individuals have twice the risk of developing hypertension and pre-eclampsia [29,30] and are four times more likely to develop gestational diabetes [31]. Diabetes, hypertension and being overweight or obese are major modifiable risk factors for a range of conditions that can increase mortality in women including maternal death [32], so the potential and need for intervention in Jamaica is significant.

Although the documentation of overweight and obesity is improving for women of reproductive age in LMICs, much less data are available on maternal obesity and its consequences in pregnancy in these settings [23].Jamaica is one of the few LMICs which provides readily available data from routine health information systems to investigate emerging non communicable conditions among maternal deaths and our study is one of a few to explore the maternal burden of obesity in a LMIC. Our study has confirmed the range of co-morbidities related to obesity among maternal deaths compared to non-obese maternal deaths, which emphasize the dangers far beyond those of an obstetric nature, including the need for continued medical care among survivors to manage these conditions over the long term.

Interpretation of our findings were limited by a number of factors related to the use of routine health information data. The conditions of obesity, diabetes and hypertension in both the JHLS and JMMS were based on single clinical parameters taken during a national demographic survey or subjective clinical judgement during pregnancy or postpartum, so it was not possible to confirm the diagnosis nor clearly distinguish between different types of diabetes or hypertension and pre-eclampsia. Some cases attributed to the pre-eclampsia syndromes may have represented pre-existing hypertension for example. Thus, there could be possible measurement error/ bias around undercounting of cases and the risk posed. We were unable to compare the outcomes in pregnancy between populations of obese pregnant women who died with those who survived, as the relevant data were only available from maternal mortality surveillance data. There is no routine assessment of nutritional status in antenatal clinics in Jamaica, underlining the need for further priority to be placed on such measurements, e.g. including BMI on all medical records. The development of a maternity databank to include individual-level data from all encounters with maternity services would rectify this situation and allow exploration of risk of mortality and obstetric morbidities associated with high BMI. Without such routine data on risk status (of antenatal women), we are also unable to calculate the case fatality rates that would accurately measure the risk, as there are no measures of fecundity for this population group compared to the general population.

It became apparent during the field work for this study that medical practitioners and the wider community did not see overweight or obesity as a ‘disease’ (and being overweight was sometimes regarded as a sign of good health) so the condition is likely to be left out as a reported contributing cause of death. On the other hand, with the ‘social normalisation’ of obesity, these causes are likely to represent the upper extreme spectrum while other cases at the lower end may be classified as normal. Thus, these findings should be interpreted as indicative. There have been global debates about the classification of obesity as a disease. In 2013, the American Medical Association agreed that obesity should be classified as a disease [33] although it is still unclear how such a definition might impact on pregnancy care. In LMICs, there have been concerted efforts over the last three decades to address care for obstetric complications leading to the direct causes of maternal deaths. Without neglecting the continuing need for emergency obstetric care, the epidemic of obesity among women of child bearing age calls for a different approach and set of interventions. Jamaica has already begun to take steps to respond to these new challenges, including upgrading the technological resources and competencies of the obstetric team, developing a cadre of feto-maternal medicine specialists and establishing high dependency units within major referral hospitals. Much more needs to be done. There are limited guidelines or capabilities in many LMICs to manage medical complications in pregnancy and the special needs of that population group. Many of these LMIC countries are struggling to deal with the direct obstetric complications, which is further enhanced by the problem of NCDs. Since this study was undertaken in Jamaica, the problem of obesity appears to have escalated, in terms of clinical presentations of mothers where maternity units are having to deal with cardiac failure during labour on a regular basis for such cases. There are also practical health system challenges such as operating theatre tables not being able to tolerate weights of women above 400lb who are needing caesarean section. These pregnancies and babies are clearly precious to these women but they are challenging these women’s overall wellbeing and the health systems that care for them.

Global consensus on the priorities to push forward can help progress in countries like Jamaica. However, this should also include health promotion strategies to improve cardiovascular fitness prior to conception. We propose two areas to address: filling the information gaps in LMICs and identifying lifestyle/preventive interventions. The data available in Jamaica clearly does not provide a full picture of the problem and the relationship of obesity with childbearing. Maternal health needs to be explored further with primary studies and better data from routine sources. Furthermore, BMI calculations for pregnant women need to be based on height, weight, and not just clinical assessments/ judgements, with BMI records incorporated in the mother’s routine antenatal records. Identifying and addressing lifestyle and dietary factors in LMICs (for example, exploring strategies to change diets traditionally suited to high physical labor to one which better accommodates the current sedentary occupations of women, targeting adolescent girls and attitudes regarding obesity as a sign of prosperity) is crucial as these may be context specific and are not necessarily transferable from industrialized to transitional settings.

## Conclusion

Many LMICs are yet to realize and therefore tackle the growing problem of overweight or obesity in their countries. Jamaica has one of the few datasets in LMIC, which allow some, albeit limited, analysis in the area of medical conditions in pregnancy. This study contributes to improving the knowledge base, identifying the gaps in information and increasing awareness of the growing problem of maternal overweight and obesity in a transitional economy. Our critical analysis in the discussion highlights a key issue in maternity care: the relatively limited data systems and health systems that are poorly responsive to emerging problems in maternity care. The high prevalence of overweight and obesity in LMICs requires culturally sensitive responses, which address the social attitudes and values and needs relevant to different settings. By publicising the lack of sophisticated data/ health information systems in LMIC, we hope to encourage action and interest in reporting and recording of medical conditions in pregnancy (as well as improvements in these health systems).

## Abbreviations

LMIC: Low and Middle-income Countries
BMI: Body Mass Index
HIV: Human Immunodeficiency Virus
JHLS: Jamaica Health and Lifestyle Survey
JMMS: Jamaica Maternal Mortality Surveillance
NCDs: non-communicable diseases
WRA: Women of reproductive age (15–49 years)
